# Synthetic Engineering of Cortical Polarity During Mitosis Using Designed Proteins

**DOI:** 10.1007/978-1-0716-4224-5_17

**Published:** 2025-01-01

**Authors:** Lara K. Krüger, Joseph L. Watson, Emmanuel Derivery

**Keywords:** Asymmetric cell division, Cell polarity, Microtubule, Mitotic spindle, Synthetic biology

## Abstract

During asymmetric cell division, cortical polarity cues drive the polarization of the microtubule cytoskeleton to ensure the generation of two daughter cells with different fates. While this a critical process for development and tissue homeostasis, the underlying molecular mechanisms orchestrating those processes are not completely understood, especially in mammals. Here, we present an assay that allows the study of the molecular mechanisms driving mammalian asymmetric cell division in a high-throughput manner by capitalizing on protein design to engineer cortical polarity of virtually any protein of interest in otherwise unpolarized mammalian culture cells.

## 1 Introduction

Cell polarity critically depends on a polarized cell cortex, where specific polarity proteins are present only on one side, forming so-called “caps.” From these cortical caps, asymmetric signaling is induced, and the intracellular space becomes polarized, with the polarization of the cell cytoskeleton and the polarized localization of cell organelles. This is critical for diverse cellular functions, in particular, for epithelial cells to maintain their function as a barrier during immune responses or asymmetric cell division.

Asymmetric cell division is a hallmark of stem cells and is the process by which one cell gives rise to two daughter cells that inherit different fate determinants and, thus, acquire distinct fates. Polar caps of conserved polarity cues induce key processes that eventually allow the generation of two daughter cells having different fates [1], in particular the profound polarization of the mitotic spindle, which rotates to align along the established polarity axis [2], and the formation of an asymmetric central spindle, where one side contains a higher microtubule density as opposed to the other [3, 4].

To examine which polarity cues are *sufficient* to drive key processes of asymmetric cell division and to delineate the downstream pathways that regulate intracellular polarization in a high-throughput manner, we developed an assay that induces cortical polarity of virtually any protein of interest in a population of otherwise unpolarized cultured mammalian cells [3]. Technically, we capitalize on a de novo-designed 2D protein polymer [5] to cluster a stably expressed transmembrane segment from the outside. This allows the formation of endocytosis-resistant clusters of the transmembrane segment, to which we target intracellular proteins of interest. Remarkably, those clusters, which are uniformly spread all over the cell surface in adhered interphase cells, coalesce into an asymmetric cortical cap on round mitotic cells, thereby polarizing specifically the protein of interest in an otherwise unpolarized cultured cell [3].

In this chapter, we describe in detail how to purify the de novo-designed polymer components from bacteria, form clusters on mouse fibroblasts, and examine the effect of asymmetric caps during metaphase using live-cell imaging or during later stages of mitosis using fixed cells.

## 2 Materials

### 2.1 Reagents Polymer Component (A (d) and B(c)-GFP) Purification

1. pET plasmids containing the designed polymer component 1 (A(d)) and component 2 (B(c)-GFP), respectively.
2. BL21 *E. coli* bacteria, stored at −80 °C.
3. TYE plates with Kanamycin.
4. 4 L of 2x TY medium with Kanamycin in four 2 L baffled flasks; 1 L per flask.
5. IPTG at 1 M stock, stored at −20 °C.
6. 200 mL Lysis buffer: 25 mM Tris, 300 mM NaCl, 1 mM DTT (or 5 mM beta-mercapto ethanol), 1 mM PMSF, 0.1 mg/mL lysozyme (or 1x anti-protease tablet) in H_2_O.
7. NiNTA purification columns.
8. 500 mL Wash buffer: 25 mM Tris, 300 mM NaCl, 1 mM DTT (or 5 mM beta-mercapto ethanol), 20 mM imidazole in H_2_O.
9. 500 mL Elution buffer: 25 mM Tris, 300 mM NaCl, 1 mM DTT (or 5 mM beta-mercapto ethanol), 500 mM imidazole in H_2_O.
10. 4−10% SDS gels.
11. Concentrators (AMICON ULTRA or equivalent; Molecular weight cut-off (MWCO) = 4kD)
12. Superdex 200 16/100 Gel filtration column.
13. 1 L Gel filtration buffer: 35 mM Tris, 150 mM NaCl, 5% Glycerol in H_2_O.

### 2.2 Equipment for A (d) and B(c)-GFP Purification

1. Ultracentrifuge with JLA 8100 & JA25.50 rotor (Beckmann; or equivalent).
2. Sonicator or homogenizer.
3. Peristaltic pump.
4. Gel filtration system.

### 2.3 Cell Lines

1. 3T3 FlpIn mouse fibroblasts stably co-expressing GBP-TM-GBP, GFP-fused to the protein of interest and iRFP670-Jupiter (microtubule marker, *see* Note 1).
2. 3T3 FlpIn mouse fibroblasts stably co-expressing GBP-TM-GBP, GFP only as control and iRFP670-Jupiter (microtubule marker) (*see* Notes 2–4).

### 2.4 Reagents and Equipment for Imaging

1. 4-well, glass-bottom imaging dishes.
2. Fibronectin at 1 mg/mL in PBS.
3. Imaging medium: Leibovitz 15 medium enriched with 10% Donor Calf Serum (DCS) and 20 mM HEPES (pH 7.5)
4. Spinning Disk confocal microscope (or equivalent).

## 3 Methods

### 3.1 Purification of A (d) and B(c)-GFP (Fig. 1)

1. Transform BL21 (DE3) E. coli with pET A(d) or pET B(c)-GFP according to manufacturer’s protocol.
2. Add one clone each to two flasks containing 25 mL 2xYE with kanamycin and grow over night at 37 °C.
3. Add each 25 mL pre-culture into 1 L of 2xYE with kanamycin and grow at 37 °C.
4. When the main cultures are at exponential growth (OD_600nm_ = ~0.6) add 0.5 mM IPTG and incubate for 3 h at 37 °C.
5. Transfer cultures into 1 L centrifuge bottles and centrifuge for 20 min at 4000 × *g* at 4 °C using a JLA8100 rotor (or equivalent).
6. Discard the supernatant and resuspend the cell pellets in lysis buffer. Use 50 mL lysis buffer for 1 L of culture (100 mL in total for 2 L of culture). *From this step on, perform all steps on ice*.
7. Sonicate the resuspended pellets, kept on ice, with 20 Watt for 5 min total ‘ON’ time, using cycles of 10 s on, 10 s off, amplitude 40%. Alternatively, a homogenizer can be used.
8. Transfer the lysed pellets into 25 mL ultracentrifuge tubes and centrifuge at 20,000 × *g* for 30 min at 4 °C.
9. Pool the supernatants and keep on ice. *In the case of B(c)-GFP the supernatant will be green*.
10. Attach the NiNTA purification column to a peristaltic pump and equilibrate according to manufacturer’s instructions with wash buffer.
11. Load the cleared supernatant.
12. Wash the column with approximately 100–200 mL of wash buffer.
13. Elute the His-tagged A(d) or B(c)-GFP by flowing elution buffer and collecting 8 fractions of 5 mL in 15 mL tubes.
14. Run 12 μL of each fraction on an SDS gel and stain with colloidal Coomassie blue.
15. Identify 2–3 of the 5 mL fractions that contain the majority of either A(d), which runs at 30 kDa, or B(c)-GFP, which runs at 70 kDa.
16. Pool those fractions.
17. Concentrate the pooled fractions to approximately 5 mL using a centrifugal concentrator with a molecular weight cut-off (MWCO) of 10 kDa.
18. Equilibrate Superdex 200 16/60 column with gel filtration (GF) buffer following the manufacturer’s instructions.
19. Run the concentrated fractions over the equilibrated Superdex 200 16/60 column.
20. Wash and elute into 96 deep well plates.
21. Run 12 μL of fractions that give significant peaks in the A_280_ absorption profile on a SDS gel to identify in which fractions A (d) or B(c)-GFP is present.
22. Pool the fractions that contain most of the protein.
23. Determine the concentration of the protein solution by measuring the absorbance at 280 nM and calculate the concentration using the extinction coefficient (A(d): ε_280_ = 21620 M^−1^ cm^−1^ ; B(c)-GFP: ε_280_ = 42800 M^−1^ cm^−1^).
24. Aliquot in 1 mL tubes and store at 4 °C. Azide can be added to prevent the growth of contaminants. The proteins are stable for at least one year.

### 3.2 Cluster Formation on Cells for Live Cell Imaging (Fig. 2)

1. Coat the imaging dish with 50 μg/mL fibronectin in PBS for 30–60 min at room temperature.
2. Add trypsinized cells stably co-expressing the transmembrane construct (GBP-TM-GBP) and the GFP-tagged protein of interest or GFP only as a control (*see* Notes 2–4).
3. Let the cells adhere for at least 30 min in the incubator at 37 °C and 5% CO_2_.
4. Add 250 μL of 0.5 μM B(c)-GFP in medium per 4-well and incubate for 1 min.
5. Wash with pre-warmed medium.
6. Add 250 μL 0.5 μM A(d) in medium per 4-well and incubate for 10 min at 37 °C (*see* Note 5).
7. Wash with pre-warmed medium.
8. Add fresh, warm medium. Optional: To increase the number of mitotic cells, synchronization can be achieved by adding 30 nM nocodazole in the medium.
9. Incubate for at least 6 h.

### 3.3 Cluster Formation on Cells for Analysis of Fixed Mitotic Cells (Fig. 2)

1. Plate the cells in a round 10 cm dish 2–3 days before the experiment so that they reach ~ 50% confluency on the day of the experiment (*see* Note 6).
2. The evening before the experiment, wash the cells with pre-warmed PBS.
3. Add 1 mL of 0.5 μM B(c)-GFP in a medium per dish and incubate for 1 min.
4. Wash with pre-warmed PBS.
5. Add 4 mL of 0.5 μM A(d) in medium per dish and incubate for 10 min at 37 °C (*see* Note 5).
6. Wash with pre-warmed PBS.
7. Add 10 mL of medium and incubate overnight at 37 °C.

### 3.4 Measuring the Effect of Cortical Polarity on Spindle Orientation Using Live Cell Imaging

**Fig. 3:**
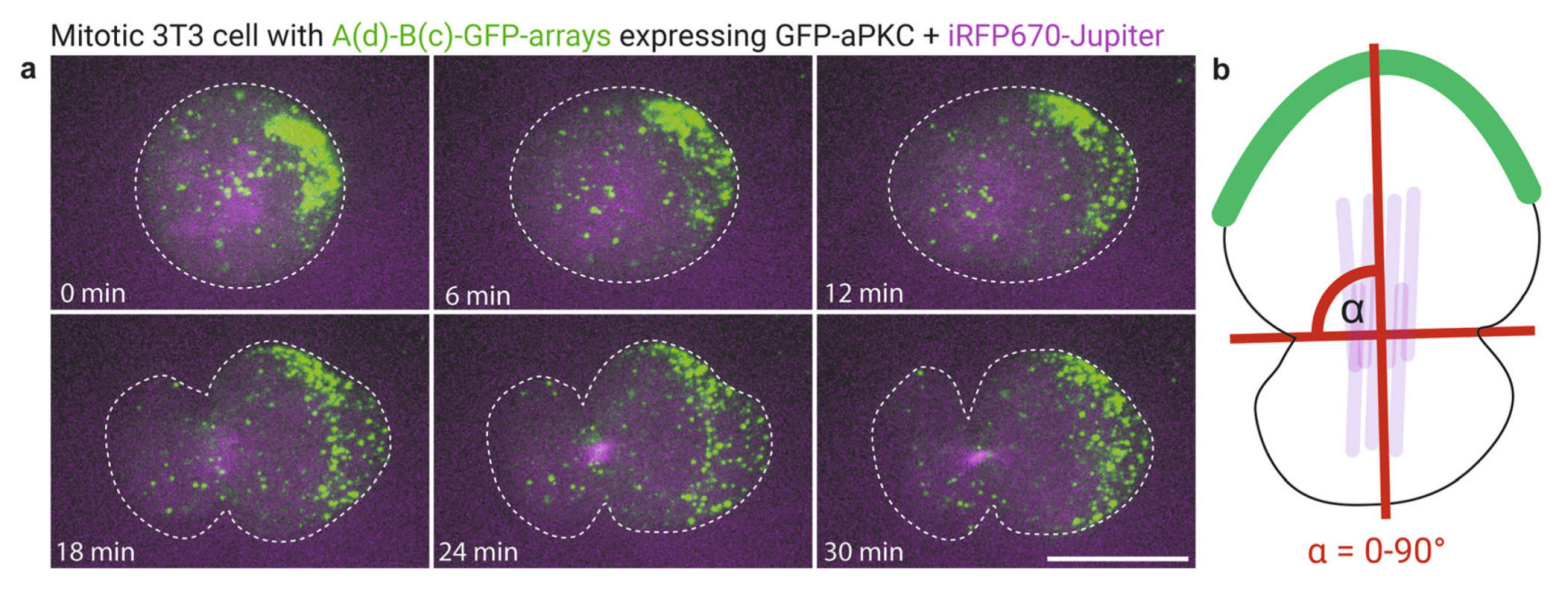
Effect of cortical aPKC caps on mitotic spindle orientation. (a) GFP caps assembled on 3T3 cells stably co-expressing iRFP670-Jupiter, GBP-TM-GBP, and GFP-aPKC. Scale bar, 10 μm. (b) The orientation angle α is the angle between the division plane and the mitotic spindle or the GFP cap

1. Pre-warm the microscope stage incubator to 37 °C.
2. Gently remove the medium.
3. Wash gently with imaging medium.
4. Add 250 μL imaging medium to each 4-well.
5. Take the dish to the microscope, find round mitotic cells with GFP caps and start imaging (*see* Note 7).
6. Caps will be visualized in the GFP channel, and the mitotic spindle can be imaged in the far-red channel using the iRFP670-jupiter microtubule marker (Fig. 3a).
7. Spindle orientation angle can subsequently be determined by computing maximum intensity projections in the preferred image analysis program (e.g., ImageJ/Fiji) and by measuring the angle between the division plane and the mitotic spindle. Alternatively, if no microtubule markers are used, the angle between the division plane and the GFP cap can be used to identify the spindle orientation angle (Fig. 3b).

### 3.5 Measuring the Effect of Cortical Polarity in Late Mitosis in Fixed Cells

1. The morning after cluster formation, coat imaging dishes with 50 μg/mL fibronectin in PBS for 30–60 min atroom temperature.
2. Wash the imaging dish with PBS and keep it at 37 °C until mitotic cells are added.
3. Wash the cells with pre-warmed PBS.
4. Add 0.5 mL of warm medium to each 10 cm dish.
5. Tap the sides of the dish hard, 5–10 taps per side.
6. Gently move the 0.5 mL of mitotic cells to the imaging dish.
7. Let the mitotic cells adhere at 37 °C for 10–20 min.
8. Gently remove the medium and wash with PBS. Then proceed with the preferred cell fixation protocol.
9. Stain with tubulin antibodies to examine the central spindle architecture upon induction of cortical polarity. In addition, the localization of any protein of interest during mitosis upon induction of cortical polarity can be examined.

## Figures and Tables

**Fig. 1:**
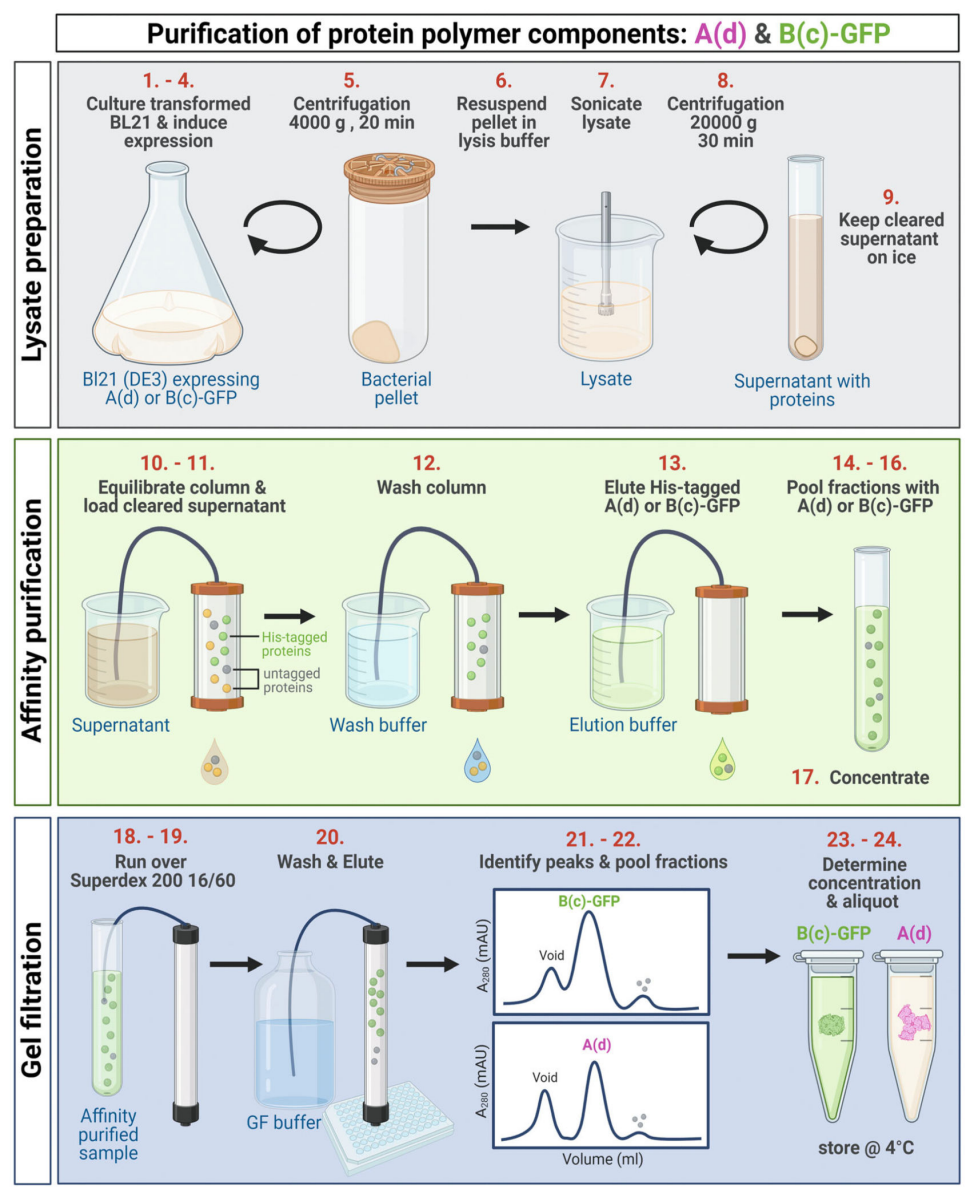
Schematic of the purification protocol of the protein polymer components A(d) and B(c)GFP

**Fig. 2:**
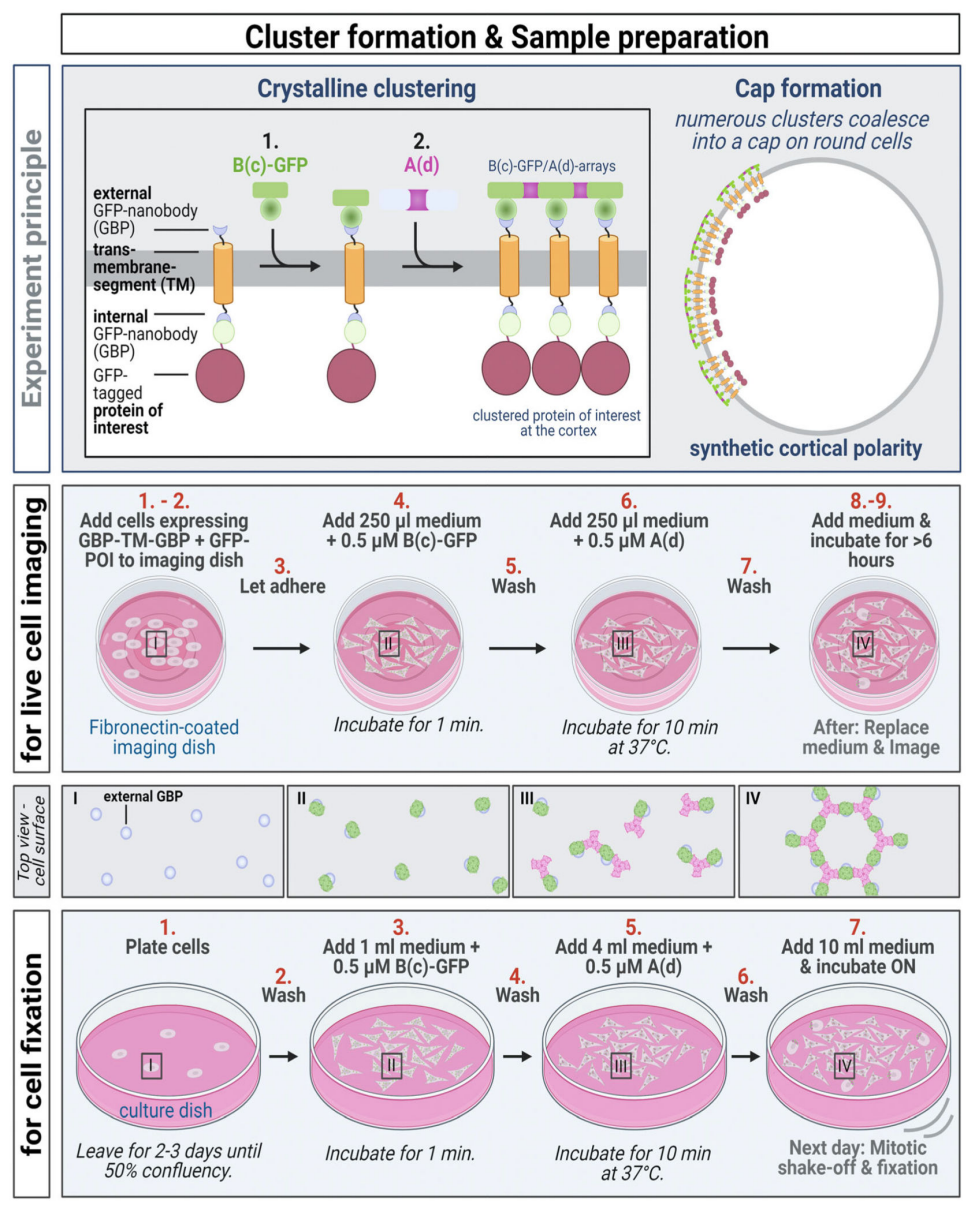
Schematic of the cluster formation on NiH3T3 cells for live cell imaging or for cell fixation after mitotic shake-off

## 4 Notes

1 In NIH-3T3 cells, iRFP670-Jupiter labels microtubules quite faintly. Microtubule dyes such as SiR-tubulin or other fluorescent mitotic spindle markers like microtubule-associated proteins could also be used.

2 While we extensively used the described protocol to induce cap formation in murine 3T3 fibroblasts, we have tested cap formation on human U2OS, mouse embryonic stem cells, and *Drosophila* S2 cells [3]. The assay can likely be adapted to a variety of other cell types, albeit slight modifications of the protocol may be required (*see* Note 5).

4 Cells with caps of GFP should always be included in every experiment as a control to rule out that the cap itself (instead of the clustered protein) influences the examined process.

5 Importantly, the concentrations of A(d) and B(c)-GFP described above have been precisely adjusted to lead to efficient cluster formation given the density of transmembrane segments achieved by stable expression of our plasmid in 3T3 cells. Different expression levels of the transmembrane segment may require adjustment of either the expression level of the transmembrane segment itself or the concentration of the designed proteins, particularly the amounts of A(d) used to induce clustering. We also recommend to always use the stable expression for the transmembrane anchors as it limits variability within the population.

6 As cluster formation is induced on the cell surface, the assay is restricted to individual cells in culture and cannot be adapted to tissues.

7 Artificial polarization with this assay can only be achieved using round cells. Indeed, cap formation is not dependent on mitosis but rather seems to depend on cell shape: cluster coalescence was only observed on round cells, irrespective of whether they are in mitosis, or not [3]. In contrast, during interphase, when 3T3 cells are not round, but rather spread, clusters are homogeneously distributed along the cell surface. Hence, the system is not adapted to reconstitute polarity in interphase.
